# Copper and cuproptosis-related genes as indictors for the diagnosis of injury time in traumatic brain injury: human cases and animal experiment

**DOI:** 10.3389/fncel.2026.1848027

**Published:** 2026-05-29

**Authors:** Rui Gu, Xiaojin Yu, Yaqing Tan, Guoliang Li, Longlong Suo, Runtao Ding, Hui Yao

**Affiliations:** 1 Faculty of Forensic Medicine, Zhongshan School of Medicine, Sun Yat-sen University, Guangzhou, Guangdong, China; 2 Guangdong Province Translational Forensic Medicine Engineering Technology Research Center, Sun Yat-Sen University, Guangzhou, Guangdong, China; 3 Department of Anesthesiology, Shengjing Hospital of China Medical University, Shenyang, Liaoning, China; 4 Center of Judicial Appraisal, Affiliated Hospital of Shandong Second Medical University, Weifang, Shandong, China; 5 Department of Forensic Medicine, School of Basic Medical Sciences, Shandong Second Medical University, Weifang, Shandong, China

**Keywords:** autopsy, cuproptosis, estimation, injury time, traumatic brain injury

## Abstract

Traumatic brain injury (TBI) is one of the most frequently encountered injuries in forensic practice. Nevertheless, a major technical bottleneck persists: there are no stable, specific indicators available for estimating the injury time of TBI in either clinical or forensic settings, which severely hinders the efficiency and accuracy of relevant case identification. Against this backdrop, the present study centers on the spatiotemporal distribution of copper ions (Cu^2+^) and the newly identified cuproptosis pathway, systematically investigating their associations with TBI progression via retrospective detection of human postmortem samples and prospective experiments on mouse TBI models. Preliminary findings revealed that within 30 days after TBI onset in humans, the copper content in the cerebral contusion area rises remarkably in a time-dependent manner, particularly in frozen-preserved cadaver samples. Concurrently, activation of the cuproptosis process was observed in the cerebral contusion region of mouse TBI models. Collectively, these findings may shed new light on the development of potential biomarkers for TBI injury time estimation. In addition, the expression levels of FDX1, DLD and SLC31A1 are applicable for inferring the time of early-stage TBI, while those of LIAS and SLC31A1 can facilitate the determination of injury time in late-stage TBI.

## Graphical abstract

**Figure:**
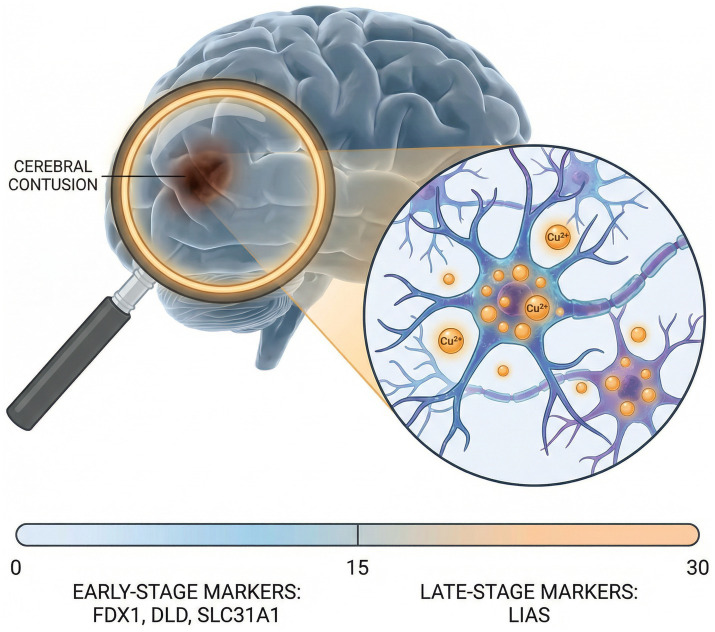
Copper ions and cuproptosis-related genes are expected to become potential biomarkers for TBI injury time estimation. The expression levels of FDX1, DLD and SLC31A1 are applicable for inferring the time of early-stage TBI, while those of LIAS and SLC31A1 can facilitate the determination of injury time in late-stage TBI.

## Research background

Traumatic Brain Injury (TBI) is defined as a pathological condition characterized by structural damage to brain tissues and consequent neurological dysfunction induced by direct external mechanical impact. It is notorious for its high incidence, disability and mortality rates, and stands as the predominant cause of death and permanent disability among young adults globally (Oft et al., 2024). Pathologically, TBI-induced brain damage encompasses two distinct categories: primary injuries directly caused by external force and secondary injuries that ensue thereafter (Jamjoom et al., 2021). Primary injuries include cerebral contusion, cerebrovascular injury, and axonal injury, among others (Kundu and Singh, 2023). Secondary injuries refer to a cascade of cellular and biochemical events triggered by the initial trauma, which represent a major contributor to post-traumatic mortality and neurological impairment; key manifestations include oxidative stress, glutamate-mediated excitotoxicity, inflammatory response, apoptosis, membrane depolarization, and calcium homeostasis imbalance (Kundu and Singh, 2023; Banoei et al., 2018). In cases of severe TBI, the insult may further result in blood–brain barrier (BBB) disruption, intracranial hematoma formation, cerebral edema, free radical overproduction, as well as the aforementioned membrane depolarization, excitotoxicity and calcium dyshomeostasis (Orešič et al., 2016; Licastro et al., 2016; Wang et al., 2016).

TBI can also perturb the homeostatic balance of copper ions (Cu^2+^) in the brain (Du et al., 2024). Disruption of the BBB and the consequent elevation of vascular permeability are likely to facilitate the infiltration of Cu^2+^ into the injured brain parenchyma. Experimental evidence has demonstrated that following TBI, oxidative stress contributes to a marked reduction in the expression of the copper chaperone Antioxidant 1 (ATOX1) in the hippocampal tissue, which in turn exacerbates the dysregulation of Cu^2+^ homeostasis (Zhao et al., 2024). Copper is an essential trace element in the human body, intricately implicated in multiple signaling pathways and biological processes associated with tumorigenesis and progression. Emerging evidence has demonstrated that cuproptosis constitutes an independent mode of regulated cell death, which is tightly linked to mitochondrial respiration and the lipoic acid (LA) metabolic pathway (Tsvetkov et al., 2022). Metabolite profiling of cells treated with copper ionophores reveals a time-dependent exacerbation of dysregulation in numerous tricarboxylic acid (TCA) cycle-associated metabolites (Chen et al., 2022). Excess Cu^2+^ primarily leads to the generation of reactive oxygen species (ROS; Huang et al., 2022). The generation of ROS products follows the principles of the Haber-Weiss reaction and the subsequent Fenton reaction leading to copper-related cuproptosis, and is thereby closely connected with ROS (Teschke and Eickhoff, 2024). Furthermore, copper treatment selectively modulates a suite of metabolic enzymes through lipoylation—a highly conserved post-translational modification (Zhou et al., 2026). Although only a limited number of proteins are lipoylated in mammalian cells, all lipoylated proteins are integral components of the TCA cycle. A key example is dihydrolipoamide S-acetyltransferase (DLAT), a subunit of the pyruvate dehydrogenase complex. Copper ions can directly bind to DLAT, promoting the disulfide bond-dependent aggregation of lipoylated DLAT. Via a genome-wide CRISPR-Cas9 screen, mitochondrial FDX1 and lipoyl synthase (LIAS) were identified as critical regulators of copper toxicity. Genetic ablation of either FDX1 or LIAS results in the accumulation of pyruvate and *α*-ketoglutarate, impairs protein lipoylation, and ultimately suppresses copper-induced cell death (Tsvetkov et al., 2019).

Metal ions exhibit relative stability during post-mortem decomposition processes. Given this inherent stability, we hypothesize that Cu^2+^ and cuproptosis—the novel regulated cell death pathway mediated by Cu^2+^—may serve as more reliable and effective indicators for estimating the injury time of TBI.

## Materials and methods

### Forensic cases and human specimens

A total of 38 TBI autopsy cases from Forensic Medicine Center of Sun Yat-sen University and the Center of Judicial Appraisal, Affiliated Hospital of Shandong Second Medical University were selected in this study (Supplementary Table 1). The selected cases all meet the following criteria: (1) Definite history of brain trauma and pathological findings of cerebral contusion; (2) No autolysis or only mild autolysis occurred in the brain tissue; (3) The interval between brain injury and biological death is less than 1 month; (4) The freezing storage time is less than 6 months. The approximately 1 cubic centimeter brain tissue at the TBI site and the adjacent normal brain tissue samples were obtained during the dissection process.

The use of autopsy cases and human samples were simultaneously approved by the Ethics Committee of Sun Yat-sen University and Shandong Second Medical University. This study was also performed in accordance with the 1964 Declaration of Helsinki.

### Animals

Male C57BL/6 N mice (9–10 weeks old, weighing 17–25 g) were purchased from Beijing Vital River Laboratory Animal Technology Co., Ltd. Mice were housed two per cage with free access to food and water. The animal room was maintained at 21 ± 1 °C with a relative humidity of 50% ± 10% and a 12-h light/dark cycle (lights on from 06:00 to 18:00).

All animal procedures were reviewed and approved by the Institutional Animal Care and Use Committee (IACUC) of Sun Yat-sen University. Experiments were performed in accordance with the National Institutes of Health Guide for the Care and Use of Laboratory Animals (NIH Publication No. 8023, revised 1978).

### Traumatic brain injury mouse model

According to previous reports (Mercurio et al., 2024), this study established the TBI mouse model by using the free fall method. Anesthetized mice were given 2% pentobarbital sodium solution by intraperitoneal injection, with an injection dose of 0.05 milliliters per 10 grams of body weight. After the anesthetic drugs take effect, cut off the hair on the head and disinfect it strictly. A 2-centimeter-long incision was made in the middle of the mouse scalp, and the periosteum at the left top of the skull was removed. The mouse was firmly fixed on the stereotactic instrument. Subsequently, a bone window with a diameter of approximately 4 millimeters was drilled 1 millimeter behind the coronal suture and 1 millimeter to the left of the midline using a bone drill to expose the dura mater. The improved free-fall method was adopted. A 30-gram weight was dropped freely from a height of 30 centimeters and struck the dura mater to simulate the situation of brain trauma. The scalp was disinfected and sutured layer by layer, disinfected, and the scalp incision was bonded with 3 M tissue glue. The exact time of the strike was recorded. In this experiment, a sham operation group (Sham group) was set up. This group of mice only underwent scalp incision and skull fenestration, without any blow to the brain tissue, which would not cause brain injury.

### Detection of Cu^2+^ in samples

Cu^2+^ levels in brain tissue samples were measured using a Cell Copper Colorimetric Assay Kit (E-BC-K775-M, Elabscience, Wuhan, China) according to the manufacturer’s instructions. Briefly, each homogenized sample was diluted 5-fold, and 50 μL of the diluted sample was used per assay. Absorbance was measured at 580 nm, and Cu^2+^ concentrations were calculated from a standard curve.

### PCR array and quantitative real-time PCR

Cells were exposed to 10^−6^ M test BPs or 0.1% DMSO (vehicle control) for 48 h, after which total RNA was isolated using TRIzol reagent (Omega, GA, United States) according to the manufacturer’s protocol. Gene expression profiling was performed using a Mouse PCR Array kit (wc-mRNA0359-M, Wcgene Biotech, Shanghai, China), as previously reported (Huang et al., 2022). Beta-actin (ACTB) and glyceraldehyde-3-phosphate dehydrogenase (GAPDH) were used as endogenous reference genes, and the stability of their expression was evaluated. Relative mRNA expression levels of target genes were calculated using the 2^−^ΔΔCt method. Ct values were normalized to ACTB, and log₂(fold-change) values were derived from 2^−^ΔΔCt; absolute log₂ ratios ≥ 1 were considered meaningful. To validate the PCR array results, quantitative real-time PCR (qRT-PCR) was performed for six selected genes using the Realplex4S qRT-PCR System (Eppendorf, Germany). Each reaction (25 μL total volume) was conducted under the following conditions: 42 °C for 60 min and 72 °C for 10 min, followed by 45 cycles of 95 °C for 10 s, 60 °C for 30 s, and 70 °C for 1 min, with a final extension at 68 °C for 7 min. Three independent biological samples were analyzed, each in technical triplicate.

### Statistical analysis

Data were analyzed using GraphPad Prism 10. All results are presented as mean ± standard deviation (SD). Normality and variance were assessed prior to statistical testing (Yao et al., 2023). Comparisons between two groups were performed using paired t-tests, and multiple-group comparisons were conducted using analysis of variance (ANOVA), as appropriate. A *p* value < 0.05 was considered statistically significant.

## Results

### In postmortem studies of human TBI cases, elevated Cu^2+^ levels have been observed in cerebral contusion regions

**Figure 1:**
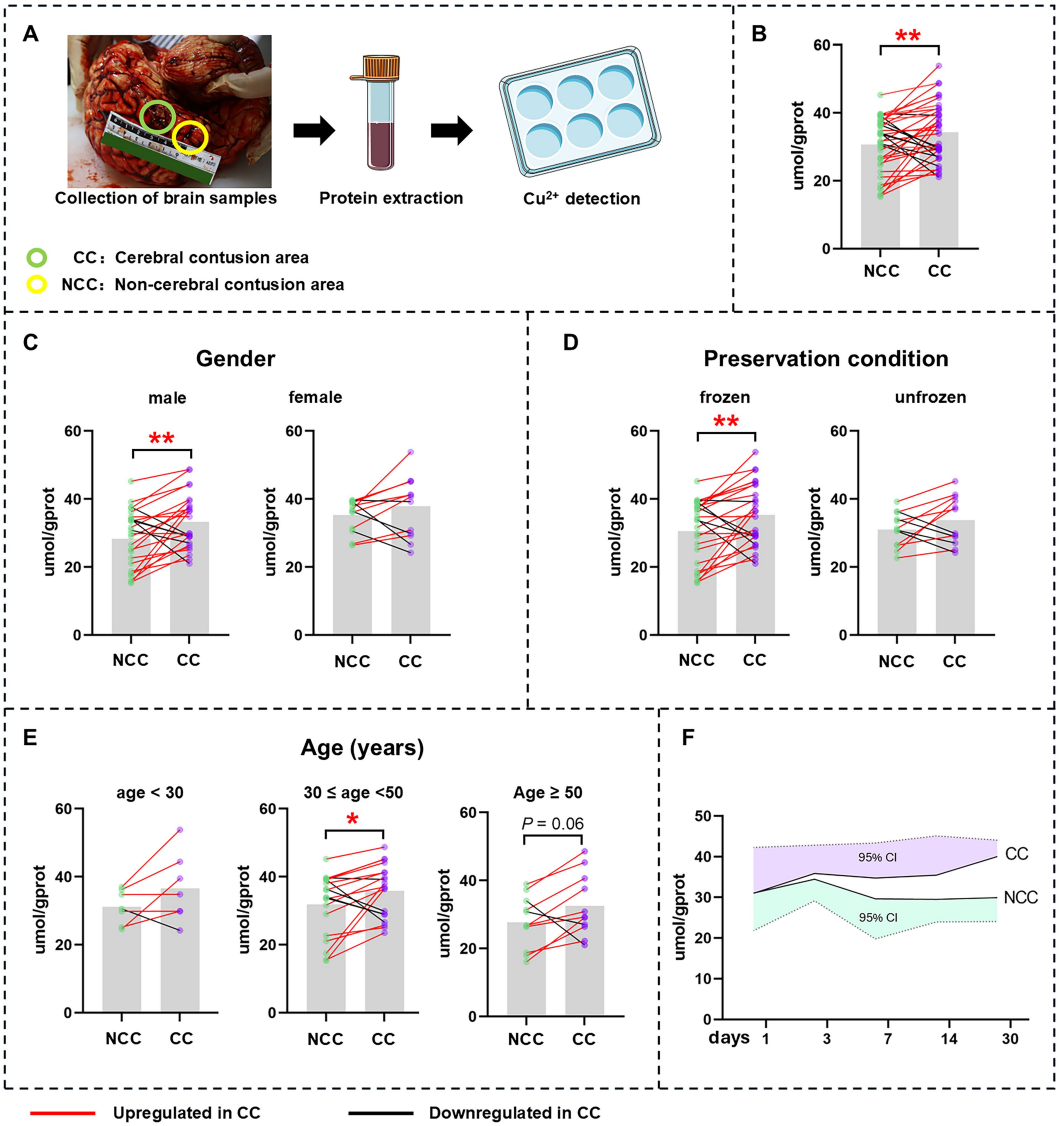
Cu^2+^ levels increased in human cerebral contusion area after TBI. **(A)** Experimental procedure and the sites for brain sample collection. **(B)** Cu^2+^ levels increased in human cerebral contusion area. **(C)** The influence of gender on the cerebral Cu^2+^ levels after TBI. **(D)** The influence of preservation condition on the cerebral Cu^2+^ levels after TBI. **(E)** The influence of age on the cerebral Cu^2+^ levels after TBI. **(F)** The pattern of changes in cerebral Cu^2+^ levels over time after TBI. **(A–E)** Statistical analysis used paired-t test, *p < 0.05, **p < 0.01. **(F)** The lines represent the average values, while the color blocks represent the 95% confidence intervals (95% CI). Human TBI cases were collected from Forensic Medicine Center of Sun Yat-sen University and the Center of Judicial Appraisal, Affiliated Hospital of Shandong Second Medical University.

To assess Cu^2+^ levels in both injured and normal areas of TBI brain tissue, a paired t-test was employed (Figure 1A). The results indicated a significant increase in Cu^2+^ concentration within the brain contusion region. Quantitatively, elevated Cu^2+^ levels were observed in the contused area compared to the healthy tissue in 23 out of 32 cases (71.88%, Figure 1B). Given variations in age, gender, and other factors among the included cases, the study subjects were further categorized into subgroups for detailed analysis.

#### Gender

When analyzed by gender, male TBI patients exhibited a significant increase in Cu^2+^ levels in the brain contusion area. In the female group, although 69.23% of cases showed higher Cu^2+^ levels in the contusion area compared to the non-contusion area, this difference was not statistically significant overall. (Figure 1C).

#### Preservation condition

In China, bodies that are not subjected to an immediate autopsy upon discovery are typically placed in mortuary refrigerators (about −20 °C) for preservation. Freezing preservation can delay the onset of corpse decomposition; however, subsequent thawing may induce structural damage to tissues and organs. In cryopreserved cadavers, the Cu^2+^ levels were elevated in contusion area compared to non- contusion area. However, this difference was not observed in non-cryopreserved cadavers. (Figure 1D).

#### Age

We divided all the cases into three groups according to age: the youth group (< 30 years), the middle-aged group (30 years ≤ age < 50 years), and the elderly group (age ≥ 50 years). Across all groups, Cu^2+^ levels in the injured brain tissue exhibited an increasing trend, however, statistical significance was observed only in the middle-aged group (*p* < 0.05). Given that the *p*-value for the elderly group is as low as 0.06, and considering the relatively small sample sizes in both the adolescent and elderly groups, we conclude that the observed changes in Cu^2+^ are likely applicable across all age groups (Figure 1E).

#### Time of injury

To demonstrate the impact of time of injury, we have plotted a line graph showing the variation of Cu^2+^ over time (Figure 1F). The longer the interval between the occurrence of TBI injury and death, the greater the increase in Cu^2+^ levels in the brain contusion area.

These results demonstrate that the alterations of Cu^2+^ levels in brain tissue following TBI exhibit a distinct temporal pattern, which is of substantial significance for the accurate estimation of injury onset time. Nevertheless, it is crucial to recognize that solely relying on Cu^2+^ content fluctuations to deduce injury timing is not universally applicable to all subgroups and has notable limitations. Therefore, efforts should be directed toward identifying potential molecular targets involved in Cu^2+^-associated physiological processes.

### The expression of genes related to cupropoptosis showed a temporal regular pattern change in the mouse model

**Figure 2:**
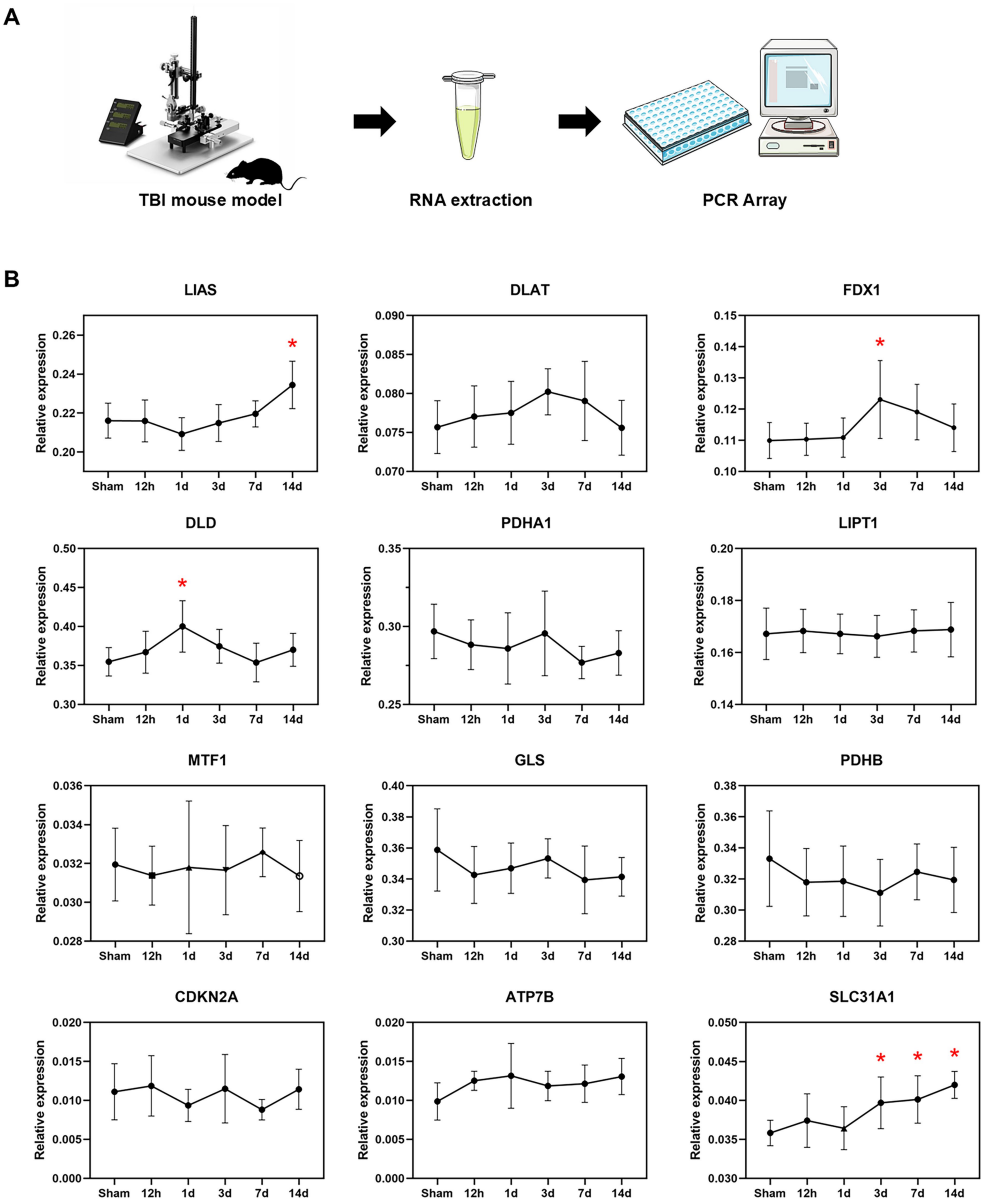
Expression of cuproptosis-related genes in TBI mouse model. **(A)** Experimental procedure. **(B)** Results of PCR Array: expression of cuproptosis-related genes in cerebral contusion areas of TBI mouse model. Statistical analysis used analysis of variance (ANOVA), *p < 0.05 compared with the Sham groups.

Based on observed alterations in cerebral copper levels postmortem, we propose that cuproptosis may serve as an indicator for estimating the time of TBI, and have developed animal models to further investigate this hypothesis (Figure 2A).

The expression of DLD in brain tissue was upregulated on day 1 post-TBI, while FDX1 expression increased on day 3 post-TBI and LIAS expression was elevated on day 14 post-TBI. In addition, the expression of SLC31A1 remained persistently upregulated from day 3 post-TBI onwards (Figure 2B).

This study demonstrates that traumatic brain injury (TBI) induces a marked elevation of Cu^2+^ levels in brain tissue, which in turn triggers the activation of the cuproptosis pathway. Both the Cu^2+^ content in the cerebral contusion area and the expression profiles of cuproptosis-related genes are of substantial significance for the accurate estimation of injury time. Specifically, the expression levels of FDX1, DLD and SLC31A1 are applicable for inferring the time of early-stage TBI, while those of LIAS and SLC31A1 can facilitate the determination of injury time in late-stage TBI.

## Discussion

The estimation of brain injury time is a crucial and challenging issue in forensic pathology research. Accurate determination of the injury time provides a basis for identifying the nature of the case. Traditional methods for estimating injury time mainly rely on visual observation and light microscopic examination of pathological changes in brain tissues following injury. However, the time window for morphological changes after injury is relatively wide, lacking clear temporal boundaries, and even characteristic morphological changes are absent in certain phases. Thus, it remains difficult to accurately estimate the elapsed time since brain injury. Recent years, forensic pathologists have begun to detect various biomarkers, including neuronal apoptosis, cortical Nrf2 protein, serum HSP70, single-stranded DNA, aiming to explore their correlation with the post-injury elapsed time (Da Rocha et al., 2005; Hausmann et al., 2004; Michiue et al., 2008; Dong et al., 2019). Additionally, several studies have investigated various serum markers to clarify the changing patterns of serum indicators after brain injury (Ondruschka et al., 2019).

In this study, Cu^2+^ was chosen as the research subject. This selection was based not only on the relative stability of metal ions but also on the gradual clarification of physiological processes such as cuproptosis in recent years. In this study, human samples have demonstrated that the Cu^2+^ concentration in cerebral contusion areas was significantly elevated in most study groups. Although no statistically significant results were observed in some subgroups, our analysis suggests that the primary cause of this phenomenon is insufficient sample size. In conclusion, these findings adequately confirm our hypothesis that Cu^2+^ serves as a relatively stable marker following TBI, with minimal influence from factors such as age, gender, and storage conditions. Although genetic analyses of human brain tissue might yield greater insights, practical constraints prevented such analyses. Concerns about genetic privacy among the relatives of the deceased led to restricting analyses to Cu^2+^ content alone.

Cuproptosis is a recently discovered mode of cell death triggered by intracellular copper overload, which operates through a mechanism distinct from all other known cell death pathways (Li and Wang, 2025). In simple terms, excess copper promotes the aggregation of lipoylated proteins and destabilization of Fe-S cluster proteins that results in proteotoxic stress and ultimately cell death, FDX1 is an upstream regulator of protein lipoylation (Dreishpoon et al., 2023). In addition, multiple genes are involved in regulating the cuproptosis process, including pro-cuproptosis genes (LIAS, LIPT1, DLD, DLAT, PDHA1, and PDHB), anti-cuproptosis genes (MTF1, GLS, and CDKN2A) and copper transporters (ATP7B and SLC31A1; Tsvetkov et al., 2022; Xie et al., 2023). Corroborating the elevated Cu^2+^ levels observed in human samples, the TBI animal model further exhibited activation of the Cu^2+^ depletion process in the cerebral cortex, where FDX1—an upstream regulator—showed increased expression in the early stage of injury. Notably, the temporal expression patterns of different genes during the cuproptosis-related physiological process render it a potential marker for identifying TBI injury time in the future.

In summary, this study, combining human samples and animal models, demonstrated that copper and cuproptosis-related genes are capable of serving as indicators for estimating the injury time in traumatic brain injury.

## Data Availability

The original contributions presented in the study are included in the article/Supplementary material, further inquiries can be directed to the corresponding authors.

## References

[ref1] Banoei M. M. Casault C. Metwaly S. M. Winston B. W. (2018). Metabolomics and biomarker discovery in traumatic brain injury. J. Neurotrauma 35, 1831–1848. doi: 10.1089/neu.2017.5326, 29587568

[ref2] Chen L. Min J. Wang F. (2022). Copper homeostasis and cuproptosis in health and disease. Signal Transduct. Target. Ther. 7:378. doi: 10.1038/s41392-022-01229-y, 36414625 PMC9681860

[ref3] Da Rocha A. B. Zanoni C. de Freitas G. R. André C. Himelfarb S. Schneider R. F. . (2005). Serum Hsp70 as an early predictor of fatal outcome after severe traumatic brain injury in males. J. Neurotrauma 22, 966–977. doi: 10.1089/neu.2005.22.966, 16156712

[ref4] Dong W. Sun Y. Cheng H. Yang B. Wang L. Jiang Z. . (2019). Dynamic cell type-specific expression of Nrf2 after traumatic brain injury in mice. Eur. J. Neurosci. 50, 1981–1993. doi: 10.1111/ejn.14399, 30828870

[ref5] Dreishpoon M. B. Bick N. R. Petrova B. Warui D. M. Cameron A. Booker S. J. . (2023). FDX1 regulates cellular protein lipoylation through direct binding to LIAS. J. Biol. Chem. 299:105046. doi: 10.1016/j.jbc.2023.105046, 37453661 PMC10462841

[ref6] Du M. Fu J. Zhang J. Zhu Z. Huang X. Tan W. . (2024). Circspna2 attenuates cuproptosis by mediating ubiquitin ligase Keap1 to regulate the Nrf2-Atp7b signalling axis in depression after traumatic brain injury in a mouse model. Clin Transl Med 14:e70100. doi: 10.1002/ctm2.70100, 39581695 PMC11586089

[ref7] Hausmann R. Biermann T. Wiest I. Tübel J. Betz P. (2004). Neuronal apoptosis following human brain injury. Int. J. Legal Med. 118, 32–36. doi: 10.1007/s00414-003-0413-4, 14625778

[ref8] Huang H. Li S. Chen B. Wang Y. Shen Z. Qiu M. . (2022). Endoplasmic reticulum-targeted polymer dots encapsulated with ultrasonic synthesized near-infrared carbon nanodots and their application for in vivo monitoring of cu(2). J. Colloid Interface Sci. 627, 705–715. doi: 10.1016/j.jcis.2022.07.095 35878461

[ref9] Jamjoom A. A. B. Rhodes J. Andrews P. J. D. Grant S. G. N. (2021). The synapse in traumatic brain injury. Brain 144, 18–31. doi: 10.1093/brain/awaa321, 33186462 PMC7880663

[ref10] Kundu S. Singh S. (2023). What happens in TBI? A wide talk on animal models and future perspective. Curr. Neuropharmacol. 21, 1139–1164. doi: 10.2174/1570159X20666220706094248, 35794772 PMC10286592

[ref11] Li Y. Wang X. (2025). A metabolic perspective on cuproptosis. Trends Endocrinol. Metab. 37, 277–287. doi: 10.1016/j.tem.2025.06.007 40681425

[ref12] Licastro F. Hrelia S. Porcellini E. Malaguti M. Di Stefano C. Angeloni C. . (2016). Peripheral inflammatory markers and antioxidant response during the post-acute and chronic phase after severe traumatic brain injury. Front. Neurol. 7:189. doi: 10.3389/fneur.2016.00189, 27853449 PMC5089971

[ref13] Mercurio D. Pischiutta F. Seminara S. Tribuzio F. Lisi I. Pasetto L. . (2024). Inhibition of mannan-binding lectin associated serine protease (MASP)-2 reduces the cognitive deficits in a mouse model of severe traumatic brain injury. J. Neuroinflammation 21:141. doi: 10.1186/s12974-024-03133-4, 38807149 PMC11134671

[ref14] Michiue T. Ishikawa T. Quan L. Li D. Zhao D. Komatsu A. . (2008). Single-stranded DNA as an immunohistochemical marker of neuronal damage in human brain: an analysis of autopsy material with regard to the cause of death. Forensic Sci. Int. 178, 185–191. doi: 10.1016/j.forsciint.2008.03.019, 18462896

[ref15] Oft H. C. Simon D. W. Sun D. (2024). New insights into metabolism dysregulation after TBI. J. Neuroinflammation 21:184. doi: 10.1186/s12974-024-03177-6, 39075578 PMC11288120

[ref16] Ondruschka B. Woydt L. Bernhard M. Franke H. Kirsten H. Löffler S. . (2019). Post-mortem in situ stability of serum markers of cerebral damage and acute phase response. Int. J. Legal Med. 133, 871–881. doi: 10.1007/s00414-018-1925-2, 30167776

[ref17] Orešič M. Posti J. P. Kamstrup-Nielsen M. H. Takala R. S. K. Lingsma H. F. Mattila I. . (2016). Human serum metabolites associate with severity and patient outcomes in traumatic brain injury. EBioMedicine 12, 118–126. doi: 10.1016/j.ebiom.2016.07.015, 27665050 PMC5078571

[ref18] Teschke R. Eickhoff A. (2024). Wilson disease: copper-mediated cuproptosis, iron-related ferroptosis, and clinical highlights, with comprehensive and critical analysis update. Int. J. Mol. Sci. 25:4753. doi: 10.3390/ijms25094753, 38731973 PMC11084815

[ref19] Tsvetkov P. Coy S. Petrova B. Dreishpoon M. Verma A. Abdusamad M. . (2022). Copper induces cell death by targeting lipoylated TCA cycle proteins. Science 375, 1254–1261. doi: 10.1126/science.abf0529, 35298263 PMC9273333

[ref20] Tsvetkov P. Detappe A. Cai K. Keys H. R. Brune Z. Ying W. . (2019). Mitochondrial metabolism promotes adaptation to proteotoxic stress. Nat. Chem. Biol. 15, 681–689. doi: 10.1038/s41589-019-0291-9 31133756 PMC8183600

[ref21] Wang J. Jin G. Yuan Z. Yu X. Li J. Qiu T. . (2016). Plasma thrombospondin-1 and clinical outcomes in traumatic brain injury. Acta Neurol. Scand. 134, 189–196. doi: 10.1111/ane.12528, 26521864

[ref22] Xie J. Yang Y. Gao Y. He J. (2023). Cuproptosis: mechanisms and links with cancers. Mol. Cancer 22:46. doi: 10.1186/s12943-023-01732-y, 36882769 PMC9990368

[ref23] Yao H. Zhang D. Yu H. Yuan H. Shen H. Lan X. . (2023). Gut microbiota regulates chronic ethanol exposure-induced depressive-like behavior through hippocampal NLRP3-mediated neuroinflammation. Mol. Psychiatry 28, 919–930. doi: 10.1038/s41380-022-01841-y, 36280756 PMC9908543

[ref24] Zhao P. Shi W. Ye Y. Xu K. Hu J. Chao H. . (2024). Atox1 protects hippocampal neurons after traumatic brain injury via DJ-1 mediated anti-oxidative stress and mitophagy. Redox Biol. 72:103156. doi: 10.1016/j.redox.2024.103156, 38640584 PMC11047792

[ref25] Zhou K. Li Z. Liu Y. Cao L. Luo H. Wang G. . (2026). Cuproptosis-based nanomedicine in cancer metastasis synergistic therapy. Acta Pharm. Sin. B 16, 1971–1990. doi: 10.1016/j.apsb.2025.11.003, 42039293 PMC13104640

