# Supplementary material for: Copper and cuproptosis-related genes as indictors for the diagnosis of injury time in traumatic brain injury: human cases and animal experiment
Source: Front Cell Neurosci. 2026 May 29;20:1848027. doi: 10.3389/fncel.2026.1848027 (PMC13259861; doi:10.3389/fncel.2026.1848027)
Supplement: Supplementary file 1 [file Table_1.DOCX]

Table 1 The concentration of copper ions in the contused and non-contused areas of human brain tissue after TBI

| Case  Number | Gender | Age (years) | The interval from injury to death | The interval between autopsy and death | Storage conditions | Cu2+ content in the non-contusion area (umol/gprot) | Cu2+ content in contusion area (umol/gprot) |
| --- | --- | --- | --- | --- | --- | --- | --- |
| 1 | male | 62 | 13days | 2days | Unfrozen | 26.57 | 37.57 |
| 2 | male | 31 | 5days | 2months | frozen | 15.33 | 36.47 |
| 3 | female | 42 | 3days | 10days | frozen | 39.68 | 39.22 |
| 4 | female | 58 | 2days | 3days | Unfrozen | 26.44 | 29.29 |
| 5 | male | 53 | 6days | 13days | frozen | 16.06 | 29.06 |
| 6 | male | 47 | 23days | 3months | frozen | 29.02 | 36.44 |
| 7 | male | 42 | 3days | 2days | Unfrozen | 33.51 | 36.05 |
| 8 | male | unclear | unclear | unclear | frozen | 37.64 | 29.34 |
| 9 | female | 55 | 4days | 1day | Unfrozen | 31.35 | 40.6 |
| 10 | male | 59 | 7days | 2months | frozen | 17.87 | 26.44 |
| 11 | male | 23 | 17days | 15days | frozen | 36.23 | 44.43 |
| 12 | male | 45 | 10days | 7days | frozen | 31.67 | 33.84 |
| 13 | male | 12 | 18days | 2days | Unfrozen | 24.63 | 39.49 |
| 14 | female | 24 | 11days | 20days | frozen | 36.95 | 53.84 |
| 15 | female | 44 | 4days | 1month | frozen | 39.16 | 26.6 |
| 16 | male | 14 | 10days | 9days | frozen | 34.79 | 34.82 |
| 17 | male | 46 | 4days | 13days | frozen | 39.12 | 41.16 |
| 18 | male | 33 | 1day | 4days | frozen | 15.55 | 23.51 |
| 19 | male | 35 | 3days | 2days | Unfrozen | 34.01 | 28.91 |
| 20 | female | 29 | 1day | 1day | Unfrozen | 30.56 | 24.23 |
| 21 | male | 66 | 1day | 11days | frozen | 34 | 21.04 |
| 22 | male | 48 | 5days | 7days | frozen | 17.49 | 38.08 |
| 23 | female | 52 | 6days | 1month | frozen | 26.82 | 30.89 |
| 24 | male | 48 | 4days | 11days | frozen | 33.71 | 28.97 |
| 25 | male | 54 | 9days | 2days | Unfrozen | 30.83 | 27.08 |
| 26 | male | 55 | 6days | 13days | frozen | 37.31 | 48.59 |
| 27 | female | 58 | 2days | 1month | frozen | 38.92 | 45.32 |
| 28 | male | 41 | 11days | 11days | frozen | 21.07 | 21.77 |
| 29 | male | 42 | 5days | 2days | Unfrozen | 22.68 | 23.09 |
| 30 | male | 29 | 8days | 4months | frozen | 25.17 | 29.87 |
| 31 | male | 51 | 6days | 2months | frozen | 18.78 | 22.23 |
| 32 | female | 48 | 5days | 2days | Unfrozen | 36.39 | 29.88 |
